# Muscle Unloading During Exercise: Comparative Effects of Conventional Oxygen, NIV, and High-Flow Therapy on Neural Drive in Severe COPD

**DOI:** 10.3390/jcm14228150

**Published:** 2025-11-17

**Authors:** Javier Sayas-Catalán, Victoria Villena Garrido, Cristina Lalmolda, Ana Hernández-Voth, Marta Corral-Blanco, Miguel Jiménez-Gómez, Laura González-Ramos, Manel Luján

**Affiliations:** 1 Hospital Universitario 12 de Octubre, 28041 Madrid, Spain; javier.sayas@salud.madrid.org (J.S.-C.); m.corralblanco@gmail.com (M.C.-B.); migueljimenezgomez@gmail.com (M.J.-G.);; 2 Facultad de Medicina, Universidad Complutense de Madrid, 28040 Madrid, Spain; 3 Corporació Sanitària Parc Taulí, 08208 Sabadell, Spain; 4 Facultad de Medicina, Universitat Autonoma de Barcelona, 08193 Barcelona, Spain

**Keywords:** chronic obstructive pulmonary disease, exercise therapy, high-flow therapy, neural drive, non-invasive ventilation

## Abstract

**Objectives**: This study aimed to evaluate how non-invasive ventilation (NIV) and high-flow nasal cannula therapy (HFT) versus conventional oxygen therapy (COT) affect neural ventilatory drive during exercise in patients with severe chronic obstructive pulmonary disease (COPD). **Methods**: We conducted an experimental, controlled study with one arm and three different conditions for the same cohort. After initial testing on conventional oxygen therapy (COT), patients exercised under NIV and HFT in sequential days and a random order. **Participants**: Twenty patients (mean age 60 years old (SD 3.9), 6 female) with severe COPD (30% women) on home NIV as a bridge to lung transplantation were enrolled in this study, with a mean FEV_1_ of 19.78% predicted and marked hyperinflation. **Protocol**: Participants performed constant-load cycling exercises at 75% maximum tolerated workload under three conditions: COT, NIV, and HFT. Neuro-respiratory drive (NRD) was measured using surface parasternal and sternocleidomastoid electromyography, and mixed ANOVA was performed to analyze repeated measures across conditions. **Results**: In total, 20 patients were included in this study. NIV demonstrated superior performance, with 60% lower NRD compared to COT (488.81 µV vs. 1180.63 µV, *p* < 0.05). HFT showed intermediate effects (807.8 µV). NIV also achieved greater reduction in respiratory rate (4.2 breaths/min), lower perceived exertion (Borg score decrease: 1.8 points), and more pronounced CO_2_ reduction (5.3 mmHg) compared to both COT and HFT. **Conclusions**: NIV significantly reduces NRD during exercise in severe COPD patients compared to HFT and COT. This supports its use as a valuable adjunct to pulmonary rehabilitation in severe COPD.

## 1. Introduction

Chronic obstructive pulmonary disease (COPD) carries a high burden of mortality and morbidity worldwide [1]. Patients with severe COPD experience exercise limitation due to multiple mechanisms, including ventilatory constraints, impaired gas exchange, and haemodynamic limitation [2]. Among these, expiratory flow limitation may be a key component of developing breathlessness and exercise intolerance [3], worsening as exercise progresses and patients attempt to increase their respiratory rate (RR) at the expense of shortening expiratory time. This situation inevitably leads to dynamic hyperinflation and the development of intrinsic end-expiratory positive pressure (iPEEP) [4,5,6,7].

The final consequence of this exercise-induced dynamic hyperinflation is the development of a “neuro-ventilatory uncoupling” [6]. Despite an increase in inspiratory effort, trying to meet the breathing demands induced by exercise, patients are unable to proportionally increase tidal volume. Thus, dyspnoea and inspiratory muscle activity (both closely linked) rise while breathing efficiency reaches a plateau, limiting exercise capacity [8].

Non-invasive ventilation (NIV) has been extensively studied as a means to improve ventilation in severe COPD by reducing neural respiratory drive uncoupling through inspiratory muscle unloading, intrinsic PEEP reduction, and increased tidal volume at rest [9,10,11] and during exercise.

Therefore, NIV has been proposed as a strategy to enhance the exercise capacity of severe COPD patients. While early reports showed conflicting results and an initial meta-analysis did not show evidence of NIV’s benefit [12], there is wide heterogenicity in terms of patient selection, protocols, outcome measures, and NIV titration. Some studies have focused on naïve NIV users, with limited, poor results [13], while others have evaluated alternative ventilatory modes [14,15] or improvements in physiological markers (for example, in muscle blood flow [16] or inflammatory profile [17,18]), ventilatory efficiency and exercise capacity [19], or long-term effects.

On the other hand, high-flow therapy (HFT) is a relatively established technique that provides high flow rates (usually >30–40 L/min) of conditioned gas (air and oxygen mixture with a selected fraction of inspired oxygen (FiO_2_), heated and humidified to 100% relative humidity) [20] that has also been studied as a tool to increase exercise capacity in COPD patients. While there is much less evidence of its benefits, and most of the studies investigating it are limited, it seems a promising tool for improving exercise capacity, with better tolerance than NIV [21].

More recent meta-analyses have demonstrated the ability of NIV to improve exercise tolerance and many physiological measures, such as oxygen consumption and dyspnoea, over HFT [22,23].

While there have been several studies identifying the physiological effects of administering NIV during exercise, the effect of HFT on NRD compared with NIV has not been directly researched. A recent study by Bonnevie et al. [24] assessed the physiological effects of HFT during exercise in severe COPD, including an exploratory evaluation of neuromechanical uncoupling, and found partial improvement with some uncertainty. Unlike previous studies using fixed NIV settings, we aim to compare the effects on neural respiratory drive (NRD) during exercise between real-time electromyography (EMG)-guided NIV with high-flow therapy (HFT) support and unsupported exercise with conventional oxygen therapy (COT). To our knowledge, this is the first study to directly compare EMG-derived NRD responses across NIV, HFT, and COT. The aim of this study was therefore to compare NRD during constant-load exercise across three different conditions: COT, respiratory support with NIV, and HFT.

## 2. Materials and Methods

### 2.1. Population

The inclusion criteria for the study consisted of patients with COPD who were on the lung transplant waiting list and already evaluated by the Lung Transplant Unit at 12 de Octubre University Hospital, with the lung transplant waiting list inclusion criteria determined according to national guidelines [25]. COPD was diagnosed according to Global Obstructive Lung Disease (GOLD) guidelines, with plethysmography confirming air trapping (a Residual Volume greater than 120% of the predicted value). Evidence of dynamic air trapping during exercise was required, confirmed by flow–volume curve analyses and the visual identification of expiratory flow limitation as previously described [26]. Additionally, patients needed to be already adapted and adherent to home non-invasive ventilation (NIV) as a bridge to transplantation. A minimum use of >4 h/day for more than 20 out of 30 days of the previous month was required.

Exclusion criteria included the presence of uncontrollable comorbidities that limited the ability to perform physical exercise (such as uncontrolled ischemic heart disease, severe pulmonary hypertension, or neuromuscular disorders), refusal to participate in the study, inability to carry out the proposed exercise protocol under baseline conditions or with ventilatory support, and the absence of reliable EMG signals for analysis.

### 2.2. Ethical and Data Management Protocol

The study protocol was approved by the Ethics Committee of Hospital 12 de Octubre (Resolution 18/025) and complied with the Spanish Organic Law on Data Protection 15/1999. All participants provided informed consent, and their inclusion in this study did not affect their status on the lung transplant waiting list.

An anonymized database, linked to clinical records through consecutive codes, was stored on a secure server within the Pneumology Department, accessible only to the principal investigator.

The trial was registered at ClinicalTrials.gov (identifier: NCT04597606).

### 2.3. Study Design

We conducted an experimental, cross-sectional, controlled study, with one arm and three different conditions (NIV, COT, HFT) for the same cohort.

### 2.4. Protocol

For the exercise testing, a semi-recumbent cycloergometer was used. The subjects were instructed to cycle with their arms relaxed along their trunk and avoid grasping the handlebars or support bars to avoid cross-contamination with pectoral muscle activity (due to gripping the handlebars) [27]. Each exercise session was planned for a maximum of 10 min of constant-load cycling, followed by a 5-min recovery (unloaded cycling at a gradually decreasing pace until stopping). Participants were strongly encouraged to continue pedaling throughout the 10-min period; however, some were unable to do so due to exertion and stopped early. Nevertheless, for all participants—regardless of whether they completed the full period without stopping pedaling, the prescribed duration of the exercise phase was set at 10 min for data analysis purposes. Testing was conducted on three separate days under the following conditions:

- COT: Patients started on their usual home oxygen flow, which was then adjusted to maintain a peripheral oxygen saturation (SpO_2_) between 92% and 96% [28]. Continuous pulse oximetry was monitored, although mean SpO_2_ values were not recorded for later analysis.
- NIV: Patients were titrated as described below, with supplemental oxygen if needed, to maintain their SpO_2_ > 92% avoiding SpO_2_ values over 96%.
- HFT (Airvo 2^®^, Fisher &Paykel, Auckland, New Zealand): A flow of 40 litres per minute was selected. Supplemental O_2_ flow was initially set—before starting exercise—to maintain an estimated FiO_2_ similar to that required to achieve an SpO_2_ > 92% with COT [29], avoiding SpO_2_ values over 96%.

Each day, before starting the exercise, each patient performed three maximum inspiratory maneuvers with simultaneous EMG recording to allow calibration and normalization. No other pulmonary function was measured during or after the exercise.

At baseline, participants underwent a “ramp test” with incremental loads up to their maximum tolerated workload, and 75% of that maximal tolerated load was selected for the tests [30,31]. Patients were already included in the exercise protocol for the lung transplantation programme, so they had all previously performed at least three sessions of exercise on a cycloergometer under COT and could thus already be considered acclimated. Three constant-load exercise tests were performed over three consecutive days. The first day was performed under COT, and at the end of the exercise testing, a 5 min trial with NIV, cycling under a free protocol for pressure titration, was performed to titrate pressures. All of the patients underwent exercise with an Astral 150^®^ ventilator (Resmed©, San Diego, CA, USA) in spontaneous/timed mode (ST mode), as it had been previously shown to be capable of providing enough pressurization support across different programmes [32]. Expiratory pressure was titrated to avoid ineffective efforts and trigger delay in online flow/pressure control, and inspiratory pressure was titrated in order to reduce neural respiratory drive (NRD) by at least 20% as measured by parasternal EMG (online EMG Root Mean Square (RMS) traces were continuously monitored to obtain that decrease, with no subsequent offline calculations in that phase).

The ≥20% visual EMG RMS peak for parasternal muscle reduction was employed exclusively as a pre-exercise titration target during a brief unloaded cycling trial to standardize NIV support (intervention step). These titration data were not analyzed as outcomes.

### 2.5. Signal Recording

During the tests, patients were connected to a recording system equipped with the following sensors:

- Electrodes placed on both sides of the second parasternal space to conduct parasternal and sternocleidomastoid surface EMG, as previously described [33].
- A combined transcutaneous CO_2_ and pulse oximetry oxygen saturation sensor (Sentec TCM^®^, Therwill, Switzerland).
- Chest and abdominal respiratory inductance plethysmography (RIP) belts (Braebon QZ-RIP, Braebon Medical Corp, Ottawa, ON, Canada) for determining inspiratory and expiratory onset.
- In the NIV condition, airflow was recorded as a raw signal from a calibrated pneumotachograph placed between the tubing and usual oronasal mask and connected to a differential pressure transducer (Powerlab Spirometer FE141, AD Instruments, Sydney, Australia) and pressure mask (Powerlab MLT844, AD Instruments, Australia).

All sensors were connected to a digital-to-analogue converter (PowerLab SP, AD Instruments, Australia), and the sampling frequency was set to 2000 Hz.

EMG signals were processed to calculate NRD as previously described [34]. Briefly, an 80 Hz high-pass filter was applied, the RMS method was employed to normalize the signals, and they were referenced to the maximum peaks obtained in an MIP maneuver. Both the peak and area under the curve (AUC) of EMG contraction were used to calculate NRD. We avoided non-respiratory tonic artefacts in the EMG by estimating the onset of inspiration from the belts’ signals. NRD was calculated as the product of the respiratory rate and both the peak and area under the peak RMS, as described by Jolley et al. [35]. Respiratory rate was determined from RIP signals, independently of the ventilator’s trigger detection, ensuring that all inspiratory efforts were accounted for.

### 2.6. Data Collection

Anthropometric variables were collected, data were taken from the last spirometry and arterial blood gas analysis, and lung function tests were performed according to current national guidelines [36], with the closest test to the exercise day selected (all within the previous 6 months).

Data were collected at different time points during the exercise test: at baseline; 1, 4, 7, and 10 min into exercise; and 3 and 5 min into the recovery period. Before each constant-load exercise, participants underwent a 5-min stabilization period under their assigned ventilatory support, and values collected at the end of this period served as baseline data for each condition.

- Respiratory variables: RR; dyspnoea perception, as measured by the BORG test scale; breathless sensation, as asked; and transcutaneously monitored CO_2_ (tcCO_2_, mmHg).
- NRD was measured as described by Jolley et al. [35]. Briefly, we choose to measure parasternal (EMGpara) and sternocleidomastoid (EMGscm) EMG signals by attaching 2 electrodes, as described previously [37]. These signals were normalized and transformed through an RMS protocol, and non-respiratory tonic artefacts in the EMG were avoided in calculations as inspiration was also signalled by the inductive plethysmography belts, and peaks were referenced to the mean baseline EMG RMS activity [38]. The RMS peak and AUC of both the EMGpara and EMGscm (µV) signals were analysed at every time step by LabChart ^®^ Software, Version 8.0 (AD Instruments, Australia).

### 2.7. Statistical Analysis

Quantitative variables were described using means and standard deviations. A general linear model with repeated measures (mixed ANOVA) was used to analyze NRD variables, including the maximum parasternal RMS value across all measurements, with the signal normalized by transmissibility—both for the peak and the AUC EMGpara signal.

The same linear model was applied to repeated measures of the maximum sternocleidomastoid RMS value, also normalized, for both the peak and the area of the EMG signal. For the ANOVA, the F value, degrees of freedom, and corresponding *p*-value were recorded to assess the statistical significance of differences across conditions.

It was considered that, in the whole-sample analysis, the global EMG signal amplitude might have been underestimated due to patients who paused during exercise. Therefore, the same mixed factorial repeated-measures ANOVA was performed on a subset of patients who did not stop pedalling during the tests.

The level of significance was set at *p* < 0.05, and statistical analyses were performed using SPSS, Version 25 (Chicago, IL, USA).

## 3. Results

### 3.1. Descriptive Analysis

During the study period, 47 patients with COPD were enrolled in the lung transplant programme. Of these, 23 were receiving home NIV and met the criteria for inclusion. One patient underwent lung transplantation before the trial commenced, and two others were excluded due to consistently unreliable EMG signals despite multiple attempts. As a result, the final study cohort consisted of 20 patients with severe COPD, 30% of whom were women. Detailed anthropometric measurements, pulmonary function test results, and arterial blood gas values are presented in Table 1.

Comparative analysis showed that ventilatory pressure parameters differed significantly between home NIV settings and exercise testing conditions. Specifically, the mean Inspiratory Positive Airway Pressure (IPAP) increased from 19.57 ± 5.02 cmH_2_O during home use to 21.93 ± 5.73 cmH_2_O during exercise (*p* < 0.01), indicating a clinically meaningful rise in pressure requirements during physical activity, as outlined in Table 1.

### 3.2. Comparative Study of Respiratory Variables

**Figure 1:**
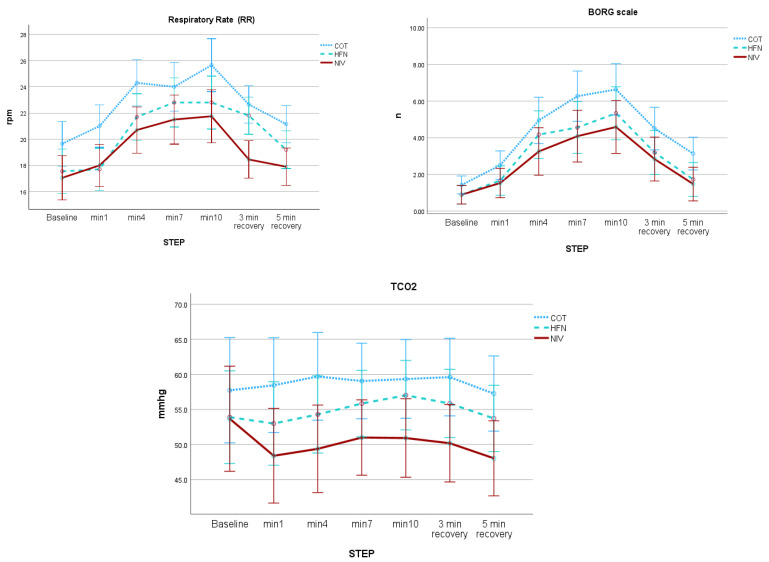
Evolution of respiratory rate (RR), dyspnea perception (Borg scale), and transcutaneous CO_2_ (tcCO_2_) during constant-load exercise under three ventilatory conditions (conventional oxygen therapy, high-flow therapy, and non-invasive ventilation). Mean ± SD values are shown for each time point (baseline; minutes 1, 4, 7, and 10 of exercise; and 3- and 5-min recovery). Results from mixed repeated-measures ANOVA show significant intra- and intercondition effects.

A comparative analysis of respiratory interventions revealed distinct physiological effects. NIV showed superior performance, with a greater reduction in RR (mean difference: 4.2 breaths/min), lower perceived exertion (Borg score decrease: 1.8 points), and a more pronounced reduction in tcCO_2_ (decrease: 5.3 mmHg) compared to both COT and HFT during exercise testing (*p* < 0.05 for all comparisons). HFT demonstrated intermediate efficacy between COT and NIV. These findings are illustrated in Figure 1, and a summary of intra- and intercondition statistics is presented in Table 2.

### 3.3. Comparative Study of Neuroventilatory Variables

During exercise, significant within-subject differences were observed in the degree of respiratory muscle activation. In the intercondition analysis, NIV resulted in the greatest muscular unloading compared to COT, with HFT showing an intermediate effect. When analyzing NRD (expressed as µV × RR) across the three exercise conditions (COT, NIV, and HFT), a mixed repeated-measures ANOVA including the full cohort demonstrated significant differences. Specifically, peak EMGpara activity was lowest with NIV, indicating the most effective reduction in respiratory muscle load during exercise. However, the analysis of the EMG area (AUC RMS for both EMGpara and EMGscm) showed no significant intercondition muscle differences, although significant within-subject changes were detected. Table 2 also summarizes NRD findings for EMGpara and EMGscm, while Table 3 reflects the average RD values for each condition

**Figure 2:**
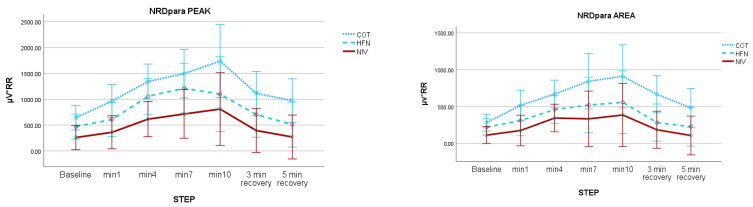

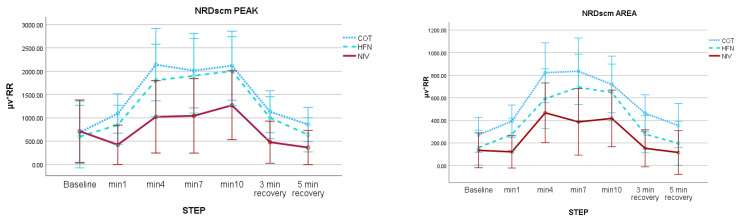
“All sample with STOPs” Peak and area values of parasternal (NRDpara) and sternocleidomastoid (NRDscm) EMG activity during constant-load exercise under three ventilatory conditions (conventional oxygen therapy, high-flow therapy, and non-invasive ventilation). Results from the full cohort are shown as mean ± SD. Mixed repeated-measures ANOVA revealed significant intra- and intercondition effects.

Finally, Figure 2 presents the whole-cohort NRD analysis.

### 3.4. Subgroup Analysis: Exercise Non-Limited Cohort (Patients Who Did Not Stop Pedalling, N = 7)

Significant time- and condition-related differences were observed in peak NRD (µV × RR) in both muscle groups analyzed, with effects observed both within and between subjects. Among the three experimental conditions, NIV was associated with the greatest reduction in respiratory muscle effort during exercise, reflected in highly significant decreases in both peak and area NRD values for the EMGpara compared with COT and HFT:

- EMGpara NRD peaks:
  ▪ Within-subject: F (6,14) = 8.970, *p* < 0.001, η^2^ = 0.79, β^−1^ = 0.99.
  ▪ Between-subject: F (2,18) = 9.116, *p* < 0.01, η^2^ = 0.50, β^−1^ = 0.94.

- EMGscm NRD peaks:
  ▪ Within-subject: F (6,33) = 23.142, *p* < 0.001, η^2^ = 0.41, β^−1^ = 0.99.
  ▪ Between-subject: F (2,33) = 4.760, *p* < 0.01, η^2^ = 0.22, β^−1^ = 0.75.

For the patients who did not stop, the differences did not reach statistical significance. When only taking normalized EMG RMS values into account (both parasternal and sternocleidomastoid, independent of RR), no significant differences were obtained between the three modalities (COT, HFT, and NIV) across the whole cohort.

## 4. Discussion

This study demonstrates that NIV significantly reduces NRD during exercise in patients with severe COPD when compared to HFT and COT. NRD, quantified as the product of RR and normalized EMGpara (EMGpara%max), was 60% lower with NIV than with COT (488.81 µV vs. 1180.63 µV, *p* < 0.05), with HFT showing intermediate effects (807.8 µV). These results are consistent with previous meta-analyses indicating the superiority of NIV over HFT in improving exercise tolerance and alleviating dyspnoea [22,39,40,41,42,43,44]. The physiological basis for this advantage lies in NIV’s ability to unload inspiratory muscles by counteracting iPEEP, reducing dynamic hyperinflation, and increasing tidal volume—mechanisms that are only modestly engaged by HFT, which primarily enhances oxygenation and comfort.

The limited impact of HFT on NRD likely reflects its inability to provide sufficient and consistent positive pressure under high ventilatory demand. Although HFT can generate a degree of airflow-dependent positive airway pressure (~4 cmH_2_O at 40 L/min), this is typically inadequate to overcome the elevated inspiratory threshold load encountered during exercise in severe COPD [21]. In contrast, NIV offers adjustable inspiratory pressures, effectively reducing inspiratory effort, as demonstrated by decreases in both EMGpara and EMGscm activity. In post hoc analyses, we observed that while some patients exhibited reductions in EMG amplitude alone, others showed minimal EMG changes but marked decreases in RR. Only the composite NRD measure (EMG × RR) revealed consistent and statistically significant differences across the cohort, underscoring the dual contribution of both respiratory muscle unloading and rate reduction to the overall ventilatory relief provided by NIV. This slowing of RR represents an additional mechanism contributing to NRD reduction and is functionally coupled with the unloading of inspiratory muscles. These findings reinforce the concept that pressure support, rather than flow delivery alone, is essential for effective respiratory muscle unloading in patients with significant expiratory flow limitation [45].

Patient characteristics likely influenced the observed efficacy of NIV. Participants presented with severe airflow limitation with a predicted first-second Forced Expiratory Volume (FEV_1_) of 19.78% and marked hyperinflation (Residual Volume/Total Lung Capacity 160%), making them particularly suitable for NIV-assisted training.

Another key contributor to NIV’s success was participants’ prior acclimatization to home NIV. This familiarity likely enhanced both tolerance and adherence, in contrast to NIV-naïve patients, who frequently experience mask-related discomfort and patient–ventilator asynchrony, often resulting in early discontinuation [13]. In our cohort, acclimatized patients achieved optimal synchrony, allowing sustained pressure delivery even during peak exercise, which likely contributed to improved alveolar ventilation and lower transcutaneous CO_2_ levels.

The use of a high-performance ventilator, with rapid pressurization capabilities (rise time < 50 ms), also played a pivotal role in matching the ventilator’s response to patients’ inspiratory demands during exercise. Unlike older or less responsive devices [13], this technology minimized flow demand and ensured consistent patient–ventilator synchrony even under rapidly changing ventilatory loads. This likely contributed to the consistent achievement of the study’s target of at least a 20% NRD reduction, a result that would be harder to obtain with suboptimal equipment. In this context, it is worth noting that proportional assist ventilation shares these physiological principles and has been shown to allow higher-intensity exercise training and greater physiological adaptation compared with conventional or unassisted exercise in severe COPD [30].

Finally, individualized inspiratory pressure titration was key to achieving physiological optimization. IPAP was adjusted prior to testing to ensure a ≥20% reduction in NRD during unloaded cycling, thereby tailoring support to each patient’s needs [13,14,46]. Comparisons of NRD reported in this study refer to the constant-load exercise phase, and real-time feedback from EMG allowed for the fine-tuning of pressure support, maximizing inspiratory muscle unloading.

The improved ventilatory pattern and lower dyspnoea with NIV suggest better exercise tolerance, in line with previous studies reporting increased endurance with ventilatory support, consistent with previous findings, such as those by Xie et al. [47], who reported a 59% improvement in dyspnoea-adjusted exercise capacity using comparable protocols. In contrast, the absence of significant benefits with HFT diverges from the review by Candia et al. [21], who observed functional improvements in patients with milder COPD. This contrast underscores HFT’s potential utility in moderate disease stages, while reinforcing the specific advantage of NIV in patients with more severe airflow limitation and hyperinflation.

These findings must also be interpreted in light of the practical demands associated with implementing NIV in clinical settings. The complete experimental setup—including EMG preparation, calibration, and transcutaneous CO_2_ stabilization—required approximately 45 min per session (for only 10 min of active, loaded exercise and 5 min of cold down), while the actual pressure titration phase lasted about 5 min and was performed by experienced personnel. This requirement poses a significant barrier in rural or resource-limited environments where access to both equipment and trained professionals may be limited. The development of automated pressure adjustment algorithms and simplified EMG monitoring techniques could play a key role in facilitating broader implementation. Moreover, future research should aim to develop simplified titration strategies that do not rely on complex monitoring. Easily measurable surrogates such as RR, dyspnoea scores, or ventilator-derived parameters could serve as practical alternatives to guide NIV initiation and adjustment in routine rehabilitation settings. But, at the same time, technological developments may offer easier approaches to these complex measurements. For example, automated expiratory flow limitation compensation may serve to titrate EPAP, or cheaper wearable devices may allow for a better understanding of respiratory muscle response during exercise [48]. Additionally, cost considerations remain an important factor, and future research should focus on evaluating whether the higher upfront costs of NIV and EMG monitoring are offset by long-term healthcare savings, particularly through reductions in hospital admissions following rehabilitation.

Accordingly, while our data provide mechanistic support—namely a robust, acute reduction in NRD during exercise—we consider it premature to recommend routine incorporation of NIV into standard pulmonary rehabilitation outside specialized settings until pragmatic trials demonstrate sustained improvements in function, symptoms, and quality of life after program completion and address real-world implementation barriers.

This study has some limitations that warrant consideration. The relatively small sample size (n = 20) constrains statistical power, and although this is comparable to previous single-centre physiological trials, it limits the generalizability of the findings to broader COPD populations. Another limitation concerns the characteristics of our study population. All participants were lung transplant candidates with very severe, “pure” COPD and minimal comorbidities, which limits the generalizability of our findings. To minimize the potential impact of comorbidities on study outcomes, all participants underwent a comprehensive pre-transplant evaluation, which, combined with the extensive pre-transplant assessment, ruled out the presence of comorbidities that confound the response to ventilatory support, and patients who are older and have milder disease or common comorbidities such as heart failure, pulmonary hypertension, or muscle dysfunction may respond differently. Furthermore, the inclusion of patients previously acclimatized to home NIV may have introduced a selection bias, potentially overestimating tolerance and benefit during exercise sessions. Another limitation concerns the HFT condition, which was applied at a fixed flow of 40 L/min without individual titration. This setting was chosen based on tolerability in severe COPD, the flow rates most commonly used in chronic home settings [49], current clinical recommendations for home HFT [50], and the potential physiological adverse effects reported with higher flows [51]. Future studies should explore EMG-guided flow titration to better define the physiological ceiling of HFT in this population. To confirm the reproducibility and external validity of these results, larger multicentre studies involving more heterogeneous patient groups and varying levels of disease severity are essential. Another important limitation is that the NIV systems used in this and previous studies are stationary and restricted to supervised settings. Attempts to develop truly portable ventilatory support for ambulation have faced major technical barriers, mainly related to battery life, device weight, and the need for high-pressure oxygen sources. Early studies using backpack-mounted ventilators [52] showed no improvement in dyspnoea or walking distance, and newer lightweight devices, such as the Philips VitaBreath, provided only intermittent support and have since been discontinued [53]. Therefore, the applicability of our findings remains limited to controlled, stationary rehabilitation environments until substantial technological advances occur.

Future research should focus on several key areas to build on these findings. First, the development of personalized ventilatory support algorithms that could enable automatic pressure adjustments during exercise, enhancing both precision and feasibility. Second, rigorous cost–benefit analyses comparing the long-term outcomes of NIV and HFT across healthcare systems with differing resource availability are needed to inform clinical and policy decisions. Third, the potential of hybrid protocols—such as sequential HFT-NIV strategies—should be investigated to determine whether they could offer an optimal balance between patient comfort and physiological efficacy, particularly in populations with variable tolerance or access to equipment.

## 5. Conclusions

This study confirms that EMG-titrated NIV significantly reduces NRD during exercise in patients with severe COPD compared to HFT and COT. These benefits are particularly evident in patients previously acclimatized to NIV and with no other comorbidities.

## Figures and Tables

**Table 1:** Patient characteristics and ventilator parameters.

Patient Characteristics	
Variable	Mean ± SD
Age (years)	60.0 ± 3.9
FEV_1_ (mL; % predicted)	580 ± 129; 19.3 ± 4.1
FVC (mL; % predicted)	2038.5 ± 739.6; 51.5 ± 18.0
RV (mL; % predicted	6240.6 ± 1242.5; 286.4 ± 28.2
TLC (mL; % predicted)	8563.9 ± 1358.9; 140.9 ± 28.2
RV/TLC ratio (%)	73.4 ± 7.8
pO_2_ (mmHg)	60.7 ± 14.2
pCO_2_ (mmHg)	51.1 ± 6.8
pH (units)	7.40 ± 0.1
**Ventilator Parameters**	
IPAP at baseline (cmH_2_O)	19.3 ± 5.0
IPAP during exercise (cmH_2_O)	21.9 ± 5.7
EPAP at baseline (cmH_2_O)	9.0 ± 2.7
EPAP during exercise (cmH_2_O)	9.5 ± 3.1

FEV_1_: Forced Expiratory Volume in 1 s (mL = absolute value; % = percent predicted); FVC: Forced Vital Capacity (mL = absolute value; % = percent predicted); RV: Residual Volume (mL = absolute value; % = percent predicted); TLC: Total Lung Capacity (mL = absolute value; % = percent predicted); RV/TLC: Residual Volume to Total Lung Capacity ratio (%); pO_2_: baseline partial pressure of oxygen in arterial blood (mmHg) under room air or home continuous oxygen; pCO_2_: baseline partial pressure of carbon dioxide in arterial blood (mmHg); pH: arterial blood pH measured under room air conditions (FiO_2_ = 0.21, Fraction of Inspired Oxygen 21%); IPAP: Inspiratory Positive Airway Pressure (cmH_2_O); EPAP: Expiratory Positive Airway Pressure (cmH_2_O); baseline: home NIV parameters; exercise: pressure values after titration, while performing constant-load cycling at 75% of maximum tolerated workload.

**Table 2:** Statistical analysis of respiratory and neural respiratory variables.

Parameter	F-Statistic	*p*-Value	Effect Size (ⴄ^2^)	Power (β^−1^)
**Respiratory Rate (RR)**				
Intrasubject	12.00	*p* < 0.05	0.20	0.88
Intersubject	6.07	*p* < 0.01	0.20	0.86
**Borg Scale**				
Intrasubject	26.08	*p* < 0.001	0.77	1.00
Intersubject	4221.59	*p* < 0.001	0.76	1.00
**TcCO_2_ (mmHg)**				
Intrasubject	1191.23	*p* < 0.001	0.96	1.00
Intersubject	26.08	*p* = 0.1	0.10	0.43
**Parasternal peak EMG**				
Intrasubject	25.95	*p* < 0.001	0.68	1
Intersubject	2.56	0.05	0.09	0.56
**Parasternal area EMG**				
Intrasubject	15.21	*p* < 0.001	0.61	0.99
Intersubject	2.52	*p* = 0.08	0.1	0.48
**SCM peak EMG**				
Intrasubject	6.53	0.08	0.2	0.67
Intersubject	2.57	0.1	0.05	0.35
**SCM area EMG**				
Intrasubject	28.04	*p* < 0.001	0.32	1
Intersubject	2.77	0.07	0.1	0.52

**Table 3:** Mean neural respiratory drive variable analysis across exercise conditions.

Parameter	Condition	Mean ± SD	95% CI
Parasternal NRD in µV (peak)	COT	1180.00 ± 200.10	781.68 to 1579.58
	NIV	488.81 ± 199.09	89.80 to 887.70
	HFT	807.80 ± 204.32	398.50 to 1217.15
SCM NRD in µV (peak)	COT	1434.24 ± 265.17	903.30 to 1965.24
	NIV	758.90 ± 265.17	227.80 to 1289.80
	HFT	1256.10 ± 265.17	725.15 to 1787.10

COT: Conventional oxygen therapy; NIV: non-invasive ventilation; NRD: neural respiratory drive; HFT: high-flow therapy; SCM: sternocleidomastoid; SD: standard deviation; CI: confidence interval.

## Data Availability

Due to the biological nature of the signals and information related to inclusion in the lung transplant list, data are not publicly available. Upon justified request, authors may share the raw flow/pressure/EMG data and other biological variable tracings.

## Abbreviations

The following abbreviations are used in this manuscript:

COPD	Chronic Obstructive Pulmonary Disease
COT	Conventional Oxygen Therapy
NIV	Non-Invasive Ventilation
HFT	High-Flow Nasal Cannula Therapy (High-Flow Therapy)
NRD	Neural Respiratory Drive
iPEEP	Intrinsic End-Expiratory Positive Pressure
EMG	Electromyography
EMGpara	Parasternal Electromyography
EMGscm	Sternocleidomastoid Electromyography
SCM	Sternocleidomastoid Muscle
RIP	Respiratory Inductance Plethysmography
SpO_2_	Peripheral Oxygen Saturation
tcCO_2_	Transcutaneous Carbon Dioxide
FEV_1_	Forced Expiratory Volume in 1 Second
FVC	Forced Vital Capacity
RV	Residual Volume
TLC	Total Lung Capacity
RV/TLC	Residual Volume to Total Lung Capacity Ratio
IPAP	Inspiratory Positive Airway Pressure
EPAP	Expiratory Positive Airway Pressure
FiO_2_	Fraction of Inspired Oxygen
MIP	Maximal Inspiratory Pressure
RMS	Root Mean Square
AUC	Area Under the Curve
RR	Respiratory Rate
SD	Standard Deviation
CI	Confidence Interval
ST mode	Spontaneous/Timed Mode (Ventilator Setting)
